# Sars-Cov-2 Infection in People with Type 1 Diabetes and Hospital Admission: An Analysis of Risk Factors for England

**DOI:** 10.1007/s13300-023-01456-8

**Published:** 2023-08-25

**Authors:** Adrian H. Heald, David A. Jenkins, Richard Williams, Rajshekhar N. Mudaliar, Amber Khan, Akheel Syed, Naveed Sattar, Kamlesh Khunti, Asma Naseem, Kelly A. Bowden-Davies, J. Martin Gibson, William Ollier, on behalf of the CVD-COVID-UK/COVID-IMPACT Consortium

**Affiliations:** 1 The School of Medicine–Manchester Academic Health Sciences Centre, The University of Manchester, Manchester, UK; 2 Department of Diabetes and Endocrinology, Salford Royal NHS Foundation Trust, Salford, UK; 3 Division of Informatics, Imaging and Data Science, Faculty of Biology, Medicine and Health, Manchester Academic Health Science Centre, University of Manchester, Manchester, UK; 4 NIHR Greater Manchester Patient Safety Translational Research Centre, The University of Manchester, Manchester, UK; 5 NIHR Applied Research Collaboration Greater Manchester, Faculty of Biology, Medicine and Health, University of Manchester, Manchester, UK; 6 School of Cardiovascular and Metabolic Health, University of Glasgow, Glasgow, UK; 7 Diabetes Research Centre, University of Leicester, Leicester, UK; 8 Department of Sport and Exercise Sciences, Musculoskeletal Science and Sports Medicine Research Centre, Manchester Metropolitan University, Manchester, UK; 9 Faculty of Science and Engineering, Manchester Metropolitan University, Manchester, UK

**Keywords:** SARS-CoV-2, COVID-19, Type 1 diabetes, Outcome

## Abstract

**Introduction:**

The severe acute respiratory syndrome coronavirus 2 (SARS-CoV-2) virus (coronavirus disease 2019 [COVID-19]) pandemic revealed the vulnerability of specific population groups in relation to susceptibility to acute deterioration in their health, including hospital admission and mortality. There is less data on outcomes for people with type 1 diabetes (T1D) following SARS-CoV-2 infection than for those with type 2 diabetes (T2D). In this study we set out to determine the relative likelihood of hospital admission following SARS-CoV-2 infection in people with T1D when compared to those without T1D.

**Methods:**

This study was conducted as a retrospective cohort study and utilised an all-England dataset. Electronic health record data relating to people in a national England database (NHS England’s Secure Data Environment, accessed via the BHF Data Science Centre's CVD-COVID-UK/COVID-IMPACT consortium) were analysed. The cohort consisted of patients with a confirmed SARS-CoV-2 infection, and the exposure was whether or not an individual had T1D prior to infection (77,392 patients with T1D). The patients without T1D were matched for sex, age and approximate date of the positive COVID-19 test, with three SARS-CoV-2-infected people living without diabetes (*n* = 223,995). Potential factors influencing the relative likelihood of the outcome of hospital admission within 28 days were ascertained using univariable and multivariable logistic regression.

**Results:**

Median age of the people living with T1D was 37 (interquartile range 25–52) years, 47.4% were female and 89.6% were of white ethnicity. Mean body mass index was 27 (standard error [SE] 0.022) kg/m^2^, and mean glycated haemoglobin (HbA1c) was 67.3 (SE 0.069) mmol/mol (8.3%). A significantly higher proportion of people with T1D (10.7%) versus matched non-diabetes individuals (3.9%) were admitted to hospital. In combined analysis including individuals with T1D and matched controls, multiple regression modelling indicated that the factors independently relating to a higher likelihood of hospital admission were: T1D (odds ratio [OR] 1.71, 95% confidence interval [CI] 1.62–1.80]), age (OR 1.02, 95% CI 1.02–1.03), social deprivation (higher Townsend deprivation score: OR 1.07, 95% CI 1.06–1.08), lower estimated glomerular filtration rate (eGFR) value (OR 0.975, 95% CI 0.974–0.976), non-white ethnicity (OR black 1.19, 95% CI 1.06–1.33/OR Asian 1.21, 95% CI 1.05–1.39) and having asthma (OR 1.27, 95% CI 1.19–1.35]), chronic obstructive pulmonary disease (OR 2.10, 95% CI 1.89–2.32), severe mental illness (OR 1.83, 95% CI 1.57–2.12) or hypertension (OR 1.44, 95% CI 1.37–1.52).

**Conclusion:**

In this all-England study, we describe that, following confirmed infection with SARS-CoV-2, the risk factors for hospital admission for people living with T1D are similar to people without diabetes following confirmed SARS-CoV-2 infection, although the former were more likely to be admitted to hospital. The younger age of individuals with T1D in relation to risk stratification must be taken into account in any ongoing risk reduction strategies regarding COVID-19/future viral pandemics.

## Key Summary Points

**Table:** 

***Why carry out the study?***
The coronavirus disease 2019 (COVID-19) pandemic revealed the vulnerability of specific population groups in relation to susceptibility to acute deterioration in their health including hospital admission and mortality. There is less data on outcomes for people with type 1 diabetes (T1D) following severe acute respiratory syndrome coronavirus 2 (SARS-CoV-2) infection.
***What was learned from this study?***
A significantly higher proportion of people living with T1D (10.7%) versus people living without diabetes (3.9%) were admitted to hospital following SARS-CoV-2 infection.
In combined analysis, including people living with T1D and people living without diabetes, multiple regression modelling indicated that the factors independently relating to a higher likelihood of hospital admission were: having T1D, social deprivation (higher Townsend deprivation score), low estimated glomerular filtration rate value, non-white ethnicity and having asthma, chronic obstructive pulmonary disease, severe mental illness or hypertension.
In this all-England study, we describe that, following confirmed infection with SARS-CoV-2, the risk factors for hospital admission for people living with T1D are similar to the general population, although people living with T1D were more likely to be admitted to hospital.
We suggest that the younger age of people living with T1D in relation to risk stratification should be taken into account in any ongoing risk reduction strategies regarding COVID-19/future viral pandemics.

## Introduction

From early 2020, the whole world was challenged by severe acute respiratory syndrome coronavirus 2 (SARS-CoV-2) virus (coronavirus disease 2019 [COVID-19]) and the associated pandemic [1]. This situation was further complicated with the successive rise of subsequent viral variants with varying clinical and transmission properties [1]. People with diabetes are known to be at a higher risk of becoming unwell and dying following SARS-CoV-2 infection, when compared to people living without diabetes [2–7], especially in association with suboptimal blood glucose control. We previously found that risk factors for hospital admission were similar to those of the general population for people diagnosed with both type 1 diabetes (T1D) and type 2 diabetes (T2D) in a city-wide UK retrospective study [4]. Specifically, in SARS-CoV-2-infected individuals with T2D, factors related to a higher admission rate included age, Townsend deprivation score, comorbidity with chronic obstructive pulmonary disease (COPD)/asthma and severe mental illness (SMI) and lower estimated glomerular filtration rate (eGFR). Metformin prescription lowered the likelihood of admission to hospital. For multivariate analysis in combined individuals with T2D, factors relating to higher likelihood of admission were having T2D, age, male gender, diagnosed COPD, diagnosed hypertension, social deprivation (higher Townsend deprivation score) and non-white ethnicity [4].

There is much less data on outcomes following SARS-CoV-2 infection for people with T1D in relation to outcomes versus those with T2D [3]. In this study we set out to determine the relative likelihood of hospital admission following SARS-CoV-2 infection in people living with T1D when compared to the general population without T1D. We also analysed the factors that may influence hospital admission in people living with T1D.

## Methods

This study was conducted as a retrospective cohort study. Analysis of electronic health record data was performed relating to people in a national England database (NHS England's Secure Data Environment, accessed via the BHF Data Science Centre's CVD-COVID-UK/COVID-IMPACT consortium) [8]. The North East—Newcastle and North Tyneside research ethics committee provided ethical approval for the CVD-COVID-UK/COVID-IMPACT research programme (REC No. 20/NE/0161) to access, within secure trusted research environments, unconsented, whole-population, de-identified data from electronic health records collected as part of patients’ routine healthcare.

The population for this cohort study comprised patients with a confirmed SARS-CoV-2 infection. The SARS-CoV-2-positive test status was taken from the COVID-19 Second Generation Surveillance System (SGSS), which contains positive COVID tests for both in-hospital testing and community testing using PCR. The exposure is whether a patient had a diagnosis of T1D prior to their COVID infection.

Each individual living with T1D (*n* = 77,392) was matched with three SARS-CoV-2-infected people living without diabetes (*n* = 223,995). Matching included the date of the positive COVID-19 test, age and gender, as recorded in the SGSS. Potential factors influencing the relative likelihood of hospital admission within 28 days were ascertained using univariable and multivariable logistic regression. We selected potential risk factors following a review of the existing literature in relation to the development of serious adverse consequences following an acute SARS-CoV-2 infection and on the basis of our previous findings [4]. Data on hospital admissions were taken from linked hospital episode statistics [9].

General practitioner data were obtained from the General Practice Extraction Service [10] Data for Pandemic Planning and Research (GDPPR) feed, held in the SDE. The follow-up period started on 1 January 2020 and ended on 1 January 2023. The project was approved and overseen by the CVD-COVID-UK/COVID-IMPACT consortium [8].

All data were "sense checked" for valid physiological ranges and internal clinical and demographic logic (date of birth, weight, height, biomarker ranges, body mass index [BMI]), as part of systematic data verification. Only BMI measurements made within 6 months of a positive COVID-19 test result were taken into account. In the final dataset we looked into potential risk variables that may enhance the possibility that persons with diabetes may be admitted to hospital after contracting COVID-19. The Townsend deprivation score [11] was utilised to describe relative social advantage/disadvantage, where greater socioeconomic disadvantage is correlated with a higher Townsend index. The term "ethnicity" was applied as defined in the 2011 census (https://www.ons.gov.uk/census/2011census). We selected potential risk factors following a review of the existing literature in relation to the development of serious adverse consequences following an acute SARS-CoV-2 infection.

We included in our analysis all individuals who had a positive test result for COVID-19 virus within 48 h of admission to take account of a COVID-19-positive status being confirmed following hospital admission.

The data extracted were then split into those with T1D and their matched controls (there was 1:3 matching) following data cleaning.

### Statistics

Missing data in digital health records is common, particularly for prescriptions and diagnoses. We hypothesised that persons for whom such information was lacking were not taking the medicine or did not have the specified condition. Due to the degree of data availability and differences in anthropometric and metabolic variables between the two groups, imputation with respect to the comparison between persons with diabetes and those without diabetes was not achievable. In other words, there was a dearth of information available for many people without diabetes. Consequently, a thorough study of the case was done. Analysis of variance (ANOVA) was used to compare continuous variables.

The key outcome was a 28-day hospital stay, and the primary exposure variable was diabetes status. All modelling was done using logistic regression. In particular models, other factors were taken into account as described below. A positive COVID-19 test result within 48 h of hospital admission was considered to be a positive test for inclusion.

We used univariate logistic regression to assess T1D versus matched people living without diabetes and looked at each relevant component in turn with a calculated odds ratio (OR) to identify potential variables linked to admission in people living with T1D. In order to determine how much the T1D OR was attenuated after all other variables were taken into account, we subsequently fitted a completely adjusted multivariable model just for T1D.

R (version 4.0.3) (R Foundation for Statistical Computing, Vienna, Austria) was used for all analyses. Unless otherwise noted, the numerical data are provided as mean ± standard deviation (SD). This analysis was performed according to a pre-specified analysis plan published on GitHub, along with the phenotyping and analysis code (https://github.com/BHFDSC/CCU040_01).

## Results

In the population examined, 77,392 people (mean [SD] age 38.9 [18.4] years) were living with T1D and had a confirmed positive COVID-19 test result, of whom 47.4% were female and 89.6% were of white ethnicity. Mean HbA1c was 67.3 (SD 19.2) mmol/mol (8.3% [SD 3.9%]) for those with T1D and 36.4 (SD 4.2) mmol/mol (5.5% [SD 0.8%]) for the controls. All people living with T1D had an HbA1c test as did 10,252 of the matched people living without diabetes (approximately 5%).

Regarding factors taken into account in this analysis, 20.5% of people living with T1D versus 9.4% of people living without diabetes had diagnosed hypertension; 1% of those with T1D had a diagnosis of enduring SMI versus 0.8% of people living without diabetes. There was no difference in smoking status between those with T1D and people living without diabetes. Metformin was prescribed for 9.6% of the people living without T1D.

A significantly higher proportion of people living with T1D (10.7%) versus matched people living without diabetes (3.9%) were admitted to hospital (for any reason) within 28 days of a positive COVID-19 test result (Table 1). The definition of a positive COVID-19 test for inclusion included a positive test result within 48 h of hospital admission.

**Table 1:** Baseline characteristics for individuals with type 1 diabetes and their controls

Variable	Controls (*n* or mean)	Controls (% or SD)	T1D population (*n* or mean)	T1D population (% or SD)
*n* individuals	223,995	100%	77,392	100%
Admission to hospital (within 28 days)	8665	3.9%	8263	10.7%
Age (years)	38.6	18.3	38.9	18.4
Townsend deprivation score (the higher the score, the greater the deprivation)	0	3.6	− 0.1	3.5
Townsend quintile (the higher the quintile, the greater the deprivation),* n*				
1	46,237	20.6%	15,612	20.2%
2	46,173	20.6%	16,372	21.2%
3	42,955	19.2%	15,548	20.1%
4	42,809	19.1%	15,136	19.6%
5	45,821	20.5%	14,724	19.0%
Latest BMI value (kg/m^2^)	27.6	6.8	27	6.1
Latest LDL cholesterol value (mmol/L)	2.9	0.9	2.4	0.9
Latest HDL cholesterol value (mmol/L)	1.4	0.4	1.5	0.4
Latest eGFR value (mL/min/1.73 m^2^)	80.6	14.2	79.7	20.3
Latest HbA1c value (mmol/mol) (%)	36.4 (5.5)	4.2 (0.8)	67.3 (8.3)	19.2 (3.9)
Latest total cholesterol value (mmol/L)	5	1.1	4.5	1.1
Patient has asthma	35,532	15.9%	12,782	16.5%
Patient has COPD	2940	1.3%	1112	1.4%
Patient has severe mental illness	1757	0.8%	761	1.0%
Patient has hypertension	20,990	9.4%	15,869	20.5%
Patient is on ACE inhibitor	9716	4.3%	12,537	16.2%
Patient is on aspirin	4098	1.8%	6182	8.0%
Patient is on clopidogrel	1922	0.9%	2361	3.1%
Patient is on metformin	347	0.2%	7418	9.6%
Hospital length of stay (days)	2.6		3.8	
Patient is a smoker	32,904	14.7%	11,731	15.2%
Ethnicity				
*White*	185,136	82.7%	69,364	89.6%
* Asian*	17,233	7.7%	3373	4.4%
*Black*	6649	3.0%	1950	2.5%
* Mixed*	4524	2.0%	1346	1.7%
* Other*	5325	2.4%	969	1.3%
* Unknown*	5128	2.3%	390	0.5%

Values in table are presented as the number (*n*) or mean as appropriate, and as the percentage or standard deviation (SD) as appropriate

*ACE* Angiotensin-converting enzyme,* BMI* body mass index,* COPD* chronic obstructive pulmonary disease,* eGFR* estimated glomerular filtration rate,* HbA1c* glycated haemoglobin,* HDL* high-density lipoprotein,* LDL* low-density lipoprotein,* T1D* type 1 diabetes

### Univariate Logistic Regression Analysis

In univariate analysis (Table 2), factors related to a greater likelihood of hospital admission in people living with T1 diabetes included older age, higher BMI, higher HbA1c, having hypertension, diagnosed SMI, COPD or asthma and being categorised in Townsend index quintiles 2–5 versus quintile 1 (quintile 1 indicates the most socially advantaged).

**Table 2:** Odds ratios and 95% confidence intervals of the univariate regression in individuals with type 1 diabetes

Variable	Odds ratio	2.5% Confidence interval	97.5% Confidence interval
Age,	1.027	1.026	1.028
Sex—male	1.044	0.997	1.093
Townsend deprivation score (the higher the score, the greater the deprivation)	1.074	1.067	1.080
Latest BMI value	1.011	1.007	1.015
Latest HbA1c value	1.017	1.016	1.018
Latest cholesterol value	0.948	0.923	0.973
Latest LDL value	0.847	0.811	0.884
Latest HDL value	0.654	0.611	0.699
Latest eGFR value	0.968	0.967	0.969
Patient has COPD	4.248	3.737	4.821
Patient has asthma	1.153	1.086	1.223
Patient has SMI	2.647	2.231	3.125
Patient has hypertension	2.714	2.586	2.849
Patient is on ACE inhibitor	1.617	1.530	1.709
Patient is on aspirin	3.532	3.319	3.756
Patient is on clopidogrel	4.380	4.005	4.787
Patient is on metformin	1.171	1.087	1.260
Townsend quartile 2	1.123	1.038	1.216
Townsend quartile 3	1.261	1.166	1.364
Townsend quartile 4	1.576	1.461	1.700
Townsend quartile 5	2.023	1.880	2.178
Patient is a smoker	1.048	0.982	1.119
Ethnicity—Black	2.279	2.034	2.548
Ethnicity—Asian	1.533	1.389	1.690
Ethnicity—Mixed	1.217	1.028	1.431
Ethnicity—Other	1.308	1.077	1.574

*SMI *Severe mental illness

### Multivariate Logistic Regression Analysis

**Fig. 1:**
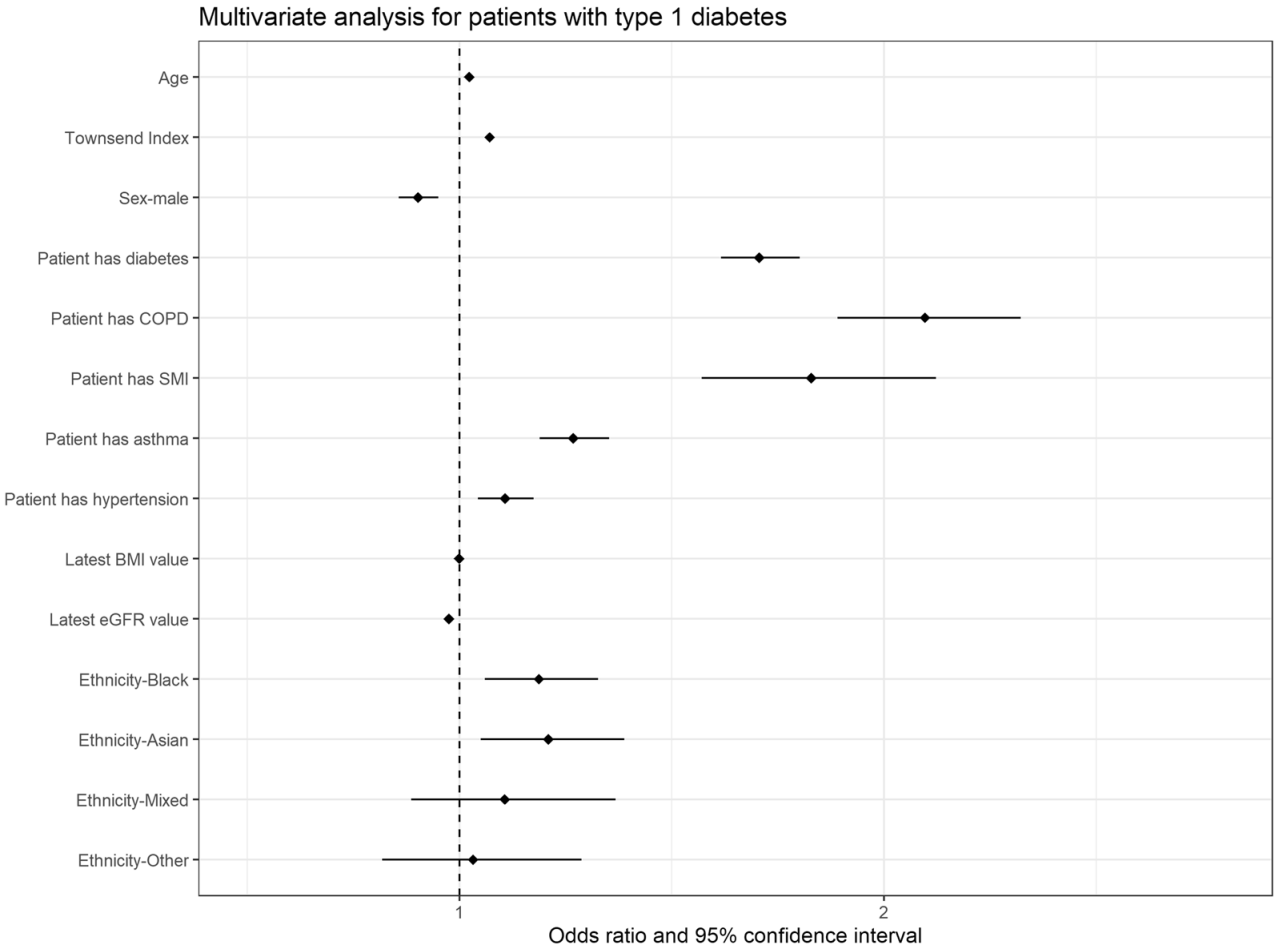
Multivariate analysis for people with type 1 diabetes versus matched controls in relation to odds ratio of hospital admission in the 28 days after a severe acute respiratory syndrome coronavirus 2 infection.* BMI* Body mass index,* COPD* chronic obstructive pulmonary disease,* eGFR* estimated glomerular filtration rate,* SMI* severe mental illness

In the combined analysis including people living with T1D and matched people living without diabetes (Fig. 1; Table 3), multiple regression modelling indicated that the factors independently relating to a higher likelihood of hospital admission were: T1D (odds ratio [OR] 1.71, 95% confidence interval [CI] 1.62–1.80]), age (OR 1.02, 95% CI 1.02–1.03), social deprivation (higher Townsend deprivation score: OR 1.07, 95% CI 1.06–1.08), lower GFR value (OR 0.975, 95% CI 0.974–0.976), non-white ethnicity (OR black 1.19, 95% CI 1.06–1.33/OR Asian 1.21, 95% CI 1.05–1.39) and having asthma (OR 1.27, 95% CI 1.19–1.35]), chronic obstructive pulmonary disease (OR 2.10, 95% CI 1.89–2.32), severe mental illness (OR 1.83, 95% CI 1.57–2.12) or hypertension (OR 1.44, 95% CI 1.37–1.52).

**Table 3:** Multivariable model combining individuals with type 1 diabetes and matched controls

Variable	Odds ratio	2.5% Confidence interval	97.5% Confidence interval
Intercept	0.163	0.132	0.201
Age	1.023	1.021	1.025
Townsend index	1.071	1.063	1.079
Sex—male	0.902	0.857	0.950
Patient has diabetes	1.706	1.616	1.802
Patient has COPD	2.096	1.891	2.322
Patient has SMI	1.829	1.571	2.123
Patient has asthma	1.268	1.189	1.352
Patient has hypertension	1.107	1.043	1.175
Latest BMI value	0.999	0.995	1.003
Latest eGFR value	0.975	0.974	0.976
Ethnicity—Black	1.187	1.060	1.327
Ethnicity—Asian	1.209	1.050	1.388
Ethnicity—Mixed	1.106	0.886	1.368
Ethnicity—Other	1.032	0.818	1.288

## Discussion

In this all-England study, we describe that, following confirmed infection with SARS-CoV-2, the risk factors for hospital admission for people living with T1D are similar to those for people without diabetes following confirmed SARS-CoV-2 infection, although people living with T1D were more likely to be admitted to hospital. The younger age of people living with T1D in relation to risk stratification must be taken into account in any ongoing risk reduction strategies regarding COVID-19/future viral pandemics, including encouragement to have the latest COVID-19 vaccination available.

Factors identified in the multivariable analysis as increasing the likelihood of hospital admission in people living with T1D included age, higher level of deprivation (Townsend deprivation index), hypertension and non-white ethnicity. In a previous study, Barron et al. reported the significant risks of SARS-CoV-2 infection in terms of adverse health consequences in T1D as well as T2D individuals [3]. These authors found that those with T1D who died were younger than non-diabetes individuals with non-Caucasian ethnicity, were more disadvantaged in terms of social situation and had a history of coronary artery disease and history of cerebrovascular disease acting as additional risk factors for death [3]. In relation to our findings, the majority of people dying following a SARS-CoV-2 infection would have been admitted to hospital in the preceding days or weeks.

When compared with our previous city wide study of hospital admission in SARS-CoV-2-infected people living with T2D [4], in the SARS-CoV-2-infected people living with T1D we did not see any influence of male sex, nor was there any influence of taking metformin. However less than 10% of people with T1D were taking metformin.

Our findings contrast with those from a population-based analysis from Belgium which showed a similar risk of hospitalisation in people living with T1D versus those living without diabetes [12]. However, in that study and in another study from the USA [13], hospitalised individuals with T1D being treated for COVID-19 had metabolic characteristics similar to those of patients with T1D who were hospitalised owing to other diagnoses, and HbA1c levels were not higher in the individuals with COVID-19. This was not the case in the present study; in particular, HbA1c was (as expected for a UK T1D group) much higher in people living with T1D versus those living without diabetes; however, it must be noted that only a small proportion of those living without diabetes had a measurement of HbA1c (approx. 5%).

In this study, we did not have access to the mode of presentation to hospital of the people living with T1D after SARS-CoV-2 infection. In a previous meta-analysis [14], the most common presentation of COVID-19 in people living with T1D included fever, dry cough, nausea and vomiting, elevated blood glucose and diabetic ketoacidosis. However, the outcomes of COVID-19 in terms of length of hospital stay, hospitalisation, intensive care unit admission, diabetic ketoacidosis rate and severe hypoglycaemia were reported in variable ways in the studies included in the meta-analysis. The authors concluded that as a consequence of the heterogeneous study populations and the presence of many limitations, more studies are warranted to look at the consequences of SARS-CoV-2 infection in people with T1D [14].

We did not analyse mortality in this study, but a higher mortality in people living with T1D compared with a population without T1D was clearly indicated in two population-based analyses from the UK [3, 15]. At particular risk of death were people with T1D who were older, had increased HbA1c levels, previous cardiovascular events (myocardial infarction, heart failure or stroke), renal functional impairment and arterial hypertension.

A significant step in primary prevention of infections is timely and appropriate vaccination. Routine vaccination against influenza may in the future be recommended in people with T1D [16] as for vaccination against COVID-19 [17]. We previously reported that adults with T1D considerably benefit from COVID-19 vaccination in terms of reduced hospitalisation [18]. We believe that the data presented here support that proposal that COVID-19 vaccination, as currently for influenza vaccination, should continue to be offered to all people living with T1D as a designated at-risk group, irrespective of age.

### Strengths/Limitations

A limitation common to all COVID-19 research is that during the first 3 months of the pandemic there was limited capacity to test for COVID-19 testing results. Consequently, the true prevalence is unknown and, specifically, prevalence for the early months of the COVID-19 pandemic can only be estimated [19]. The definition of a positive COVID-19 test result for inclusion included a positive test within 48 h of hospital admission. This has the potential to augment the association between a COVID-19 positive test result and hospital admission, but this would be expected to affect T1D and people living without diabetes in a similar way. Thus, there is the likelihood of there being an underestimate of the total number of COVID-19-positive test results for the groups studied. The purpose of our study was to explore the risk factors for admission following COVID-19 infection in someone with T1D. We accept that the decision to admit is ultimately a clinician-based decision and this is a further limitation of the study.

We used the 28-day hospital admission rate and mortality rate in comparison to Office of National Statistics (ONS) results [20]. However, there is no reason to suspect that this would affect people with diabetes versus those without diabetes differently. Another limitation is that we were not able to determine the primary diagnosis on admission, such as diabetic ketoacidosis. We accept that the proportion of people living without diabetes with a measured HbA1c or BMI was around 5% of the total number. However, we do not feel that this would have materially changed the results.

We accept that there have been other publications in this area and we acknowledge this in the manuscript. However, we believe that the link between T1D and COVID-19 in England utilising data up to early 2023 has not been previously explored. The replication of results in different data and different settings is a critical part of research.

The strengths of this study include its utilisation of a national database and that by matching our cohort on the date of a positive coronavirus test result, as well as with age and sex, we are able to correct for this and focus on the differences between the diabetes T1D cohort and the general population.

## Conclusion

In this all-England study, we describe that following confirmed infection with the COVID-19 virus, the risk factors for hospital admission for people living with T1D are similar to those for people without diabetes following confirmed SARS-CoV-2 infection, although people living with T1D were more likely to be admitted to hospital. This, and the younger age of people living with T1D in relation to risk stratification, must be taken into account in any ongoing risk reduction strategies regarding COVID-19/future viral pandemics.
