# Correction to: Sars-Cov-2 Infection in People with Type 1 Diabetes and Hospital Admission: An Analysis of Risk Factors for England

**DOI:** 10.1007/s13300-023-01483-5

**Published:** 2023-10-05

**Authors:** Adrian H. Heald, David A. Jenkins, Richard Williams, Rajshekhar N. Mudaliar, Amber Khan, Akheel Syed, Naveed Sattar, Kamlesh Khunti, Asma Naseem, Kelly A. Bowden-Davies, J. Martin Gibson, William Ollier, on behalf of the CVD-COVID-UK/COVID-IMPACT Consortium

**Affiliations:** 1 The School of Medicine–Manchester Academic Health Sciences Centre, The University of Manchester, Manchester, UK; 2 Department of Diabetes and Endocrinology, Salford Royal NHS Foundation Trust, Salford, UK; 3 Division of Informatics, Imaging and Data Science, Faculty of Biology, Medicine and Health, Manchester Academic Health Science Centre, University of Manchester, Manchester, UK; 4 NIHR Greater Manchester Patient Safety Translational Research Centre, The University of Manchester, Manchester, UK; 5 NIHR Applied Research Collaboration Greater Manchester, Faculty of Biology, Medicine and Health, University of Manchester, Manchester, UK; 6 School of Cardiovascular and Metabolic Health, University of Glasgow, Glasgow, UK; 7 Diabetes Research Centre, University of Leicester, Leicester, UK; 8 Department of Sport and Exercise Sciences, Musculoskeletal Science and Sports Medicine Research Centre, Manchester Metropolitan University, Manchester, UK; 9 Faculty of Science and Engineering, Manchester Metropolitan University, Manchester, UK

**Correction to: Diabetes Ther** 10.1007/s13300-023-01456-8

The article “Sars-Cov-2 Infection in People with Type 1 Diabetes and Hospital Admission: An Analysis of Risk Factors for England”, written by Adrian H. Heald, David A. Jenkins, Richard Williams, Rajshekhar N. Mudaliar, Amber Khan, Akheel Syed, Naveed Sattar, Kamlesh Khunti, Asma Naseem, Kelly A. Bowden-Davies, J. Martin Gibson, William Ollier, on behalf of the CVD-COVID-UK/COVID-IMPACT Consortium was originally published electronically on the publisher’s Internet portal (currently SpringerLink) on August 25, 2023, without open access. Now, the article is updated with open access as This article is licensed under a Creative Commons Attribution 4.0 International License, which permits use, sharing, adaptation, distribution and reproduction in any medium or format, as long as you give appropriate credit to the original author(s) and the source, provide a link to the Creative Commons licence, and indicate if changes were made. The images or other third party material in this article are included in the article’s Creative Commons licence, unless indicated otherwise in a credit line to the material. If material is not included in the article’s Creative Commons licence and your intended use is not permitted by statutory regulation or exceeds the permitted use, you will need to obtain permission directly from the copyright holder. To view a copy of this licence, visit http://creativecommons.org/licenses/by/4.0/.

The original article has been corrected.

